# Temporal changes in circulating miRNAs following gestational diabetes diagnosis: a pilot longitudinal study

**DOI:** 10.1016/j.jcte.2026.100448

**Published:** 2026-06-13

**Authors:** Ying Gu, Yu Chen, Jingyang Li, Lingli Hu, Yajie Gao, Hua Gong, Qi Chen

**Affiliations:** a Department of Obstetrics, Wuxi Maternity and Child Health Care Hospital, Affiliated to Jiangnan University, Wuxi, China; b Department of Medical Laboratory, Wuxi Maternity and Child Health Care Hospital, Affiliated to Jiangnan University, Wuxi, China; c Department of Obstetrics and Gynaecology, The University of Auckland, Auckland, New Zealand

**Keywords:** GDM, longitudinal study, miRNAs, C19MC, Prediction, Biomarkers

## Abstract

Currently, no clinical early predictive biomarkers of GDM exist. A key challenge is that many candidate biomarkers fluctuate during pregnancy. Previous studies with a single time point identified several miRNAs in early pregnancy associated with insulin sensitivity between 24 and 29 weeks of gestation, suggesting they serve as potential predictive biomarkers for GDM. However, the lack of longitudinal data limits our understanding of how these miRNAs behave over time. We conducted a longitudinal pilot study to evaluate the patterns of ten miRNAs, previously identified as potential early predictors of GDM. Blood samples were collected from 18 GDM women and 18 healthy pregnancies at 4 time points (GDM diagnosis, 28 weeks, 32 weeks, and one day before delivery). The levels of four placenta-specific C19MC miRNAs (miRNA-519d-5p, miRNA-512-3p, miRNA-516a-5p, and miRNA-517-5p) and six non-C19MC miRNAs (miRNA-141-3p, miRNA-143-3p, miRNA-218-5p, miRNA-221-3p, miRNA-483-5p, and miRNA-489-3p) were measured. The relative expressions of four C19MC miRNAs were significantly increased in GDM women at diagnosis, at 28 weeks of gestation, and one day before delivery. The relative expression of non-C19MC miRNA-221-3p was significantly decreased across all time points. The expression of non-C19MC miRNA-143-3p increased significantly at diagnosis and 28 weeks, and then declined before delivery. In contrast, the levels of non-C19MC miRNA-483-5p, miRNA-218-5p, and miRNA-489-3p were significantly decreased across all time points. No changes in miRNA-141-3p level were observed. Our pilot longitudinal study supports the predictive potential of C19MC miRNAs (miRNA-512-3p, miRNA-516a-5p, miRNA-517-5p) and non-C19MC miRNA-221-3p, which were previously identified as having elevated levels in early gestation.

## Highlights

• The temporal changes in circulating miRNAs in GDM have not been fully investigated.
• Placental-specific and non-placental-specific miRNAs were longitudinally measured.
• C19MC miRNAs, miRNA-512-3p, miRNA-516a-5p, miRNA-517-5p can serve as predictive markers.
• Non-C19MC, miRNA-221-3p can serve as a predictive marker of GDM.

## Introduction

Gestational diabetes mellitus (GDM) is a common pregnancy complication diagnosed after 24 weeks of gestation, affecting approximately 10 to 15% of all pregnancies [1]. It is associated with adverse pregnancy outcomes and long-term health consequences for affected women [2]. Increasing evidence suggests that the alterations of microRNAs (miRNAs) present in maternal circulating extracellular vesicles (EVs) may serve as early biomarkers for predicting GDM (reviewed in [3]). MiRNAs, a class of non-coding RNAs, play a significant role in regulating gene expression and are involved in numerous physiological and pathological processes.

EVs are membrane-encapsulated particles released by all types of cells during physiological and pathological conditions [4], [5], and carry many regulatory RNAs, including miRNAs, functional proteins, DNA, and lipids [6]. During human pregnancy, a large amount of EVs are released from placental trophoblast into maternal circulation, and these EVs contain multiple specific miRNAs clustered on chromosome 19 (C19MC miRNAs) [7], which are unique to primate human placentae [8]. These C19MC miRNAs contribute to maternal adaptation and immune modulation during pregnancy (reviewed in [9]). Therefore, changes in EV-associated miRNAs may reflect abnormal maternal adaptation or placental dysfunction, potentially contributing to complications, such as GDM.

Several studies have reported altered levels of specific miRNAs in circulating EVs as early as 6 and 15 weeks of gestation in women who later developed GDM. These miRNAs included miRNA-520 h, miRNA-1323, miRNA-27a-5p, miRNA-93-5p, and miRNA-33a-5p [10], [11], [12], which potentially regulate glucose metabolism or insulin resistance [13] or pancreatic β-cell function [12]. Additionally, other studies using RNA sequencing have identified several miRNAs in the circulation during early pregnancy, including C19MC miRNAs (such as miRNA-512-3p and miRNA-519d-5p) and non-C19MC miRNAs (such as miRNA-141-3p and miRNA-143-3p), which are specifically associated with insulin sensitivity between 24 and 29 weeks of gestation [14]. High levels of these specific miRNAs are considered key contributors to metabolic disorders, such as insulin resistance. Given that impaired insulin sensitivity and secretion contribute to the development of GDM [15], these findings suggested a potential predictive value in measuring circulating levels of these miRNAs. However, most existing studies have assessed miRNA expression at a single time point. The lack of longitudinal data limits our understanding of the temporal dynamics of miRNA expression in GDM.

Moreover, maternal physiological adaptations or gestational changes may cause natural fluctuations in miRNA levels, potentially leading to false-positive associations when measurements are taken at a single gestational stage. If miRNA levels change around the time of GDM onset or progression, it becomes challenging to determine whether these miRNAs are involved in disease initiation, reflect ongoing metabolic dysfunction, or are simply markers of normal gestational adaptation. These limitations may partly explain why no circulating miRNA biomarkers have yet been translated into clinical practice for the early prediction of GDM.

To address this gap, we conducted the first longitudinal pilot study to evaluate the expression patterns of ten miRNAs, including four C19MC and six non-C19MC miRNAs, previously identified as potential early predictors of GDM at a single time point [14], [16]. We selected all four C19MC miRNAs and randomly selected six of the 14 non-C19MC miRNAs identified in a previous study [14]. We analysed samples collected at the time of GDM diagnosis, at 28 and 32 weeks of gestation, and one day before delivery to assess how miRNA levels change over time in women with GDM compared with those in pregnant women without complications. The aim of this study is to provide deeper insights into the temporal dynamics of miRNA expression and their potential roles in disease progression and maternal adaptation.

## Methods

This study received approval from the Ethics Committee of Wuxi Maternity and Child Health Hospital, China (reference number 2023010628–16). Blood samples were collected from participants after they had signed a consent form.

### Study participants

This longitudinal study involved the prospective collection of blood samples from women diagnosed with GDM (*n* = 18) and healthy pregnant women (n = 18). The sampling time points included GDM diagnosis (or 24 weeks for controls), at 28 and 32 weeks of gestation, and one day before delivery. Participants in either group who developed pregnancy complications, such as preeclampsia or foetal growth restriction (FGR), after these time points were excluded from the final analysis. Furthermore, as miRNAs measured in this study are specifically associated with insulin sensitivity [14], the final GDM women (*n* = 18) included in this study did not receive insulin treatment and were instead managed with lifestyle interventions after diagnosis. The clinical details of women with GDM are summarized in Table 1. Serum was collected by centrifugation at 3000 ×*g* for 5 min and stored at −70 °C for later RNA isolation.

**Table 1:** Clinical parameters of the study cohort.

	GDM (n = 18)	Healthy controls (n = 18)	*P* value
Maternal age (years, mean/SD)	31 ± 2.6	29 ± 3.7	0.129
Gravidity (n, %)			>0.999
1	10 (56%)	10 (56%)	
≥2	8 (44%)	8 (44%)	
Parity (n, %)			>0.999
0	13 (72%)	13 (72%)	
≥1	5 (28%)	5 (28%)	
Birthweight (g, mean/SD)	3236 ± 408	3131 ± 353	0.443
Delivery week (mean/SD)	39^+3^ ± 1^+2^	39^+3^ ± 0.7^+2^	0.879

Parity: excluding the current pregnancy.

GDM was defined as circulating blood glucose levels greater than 5.1 mmol/L at 0 h of 75 g-OGTT (fasting glucose levels), and/or blood glucose levels greater than 10.0 mmol/L at 1 h of 75 g-OGTT, and/or blood glucose levels greater than 8.5 mmol/L at 2 h of 75 g-OGTT, between 24 and 28 weeks of gestational age, following the International Association of Diabetes and Pregnancy Study Groups (IADPSG) guideline [17]. A healthy pregnancy was defined as the absence of maternal and foetal complications until delivery.

### RNA extraction and miRNA measurement

**Fig. 1:**
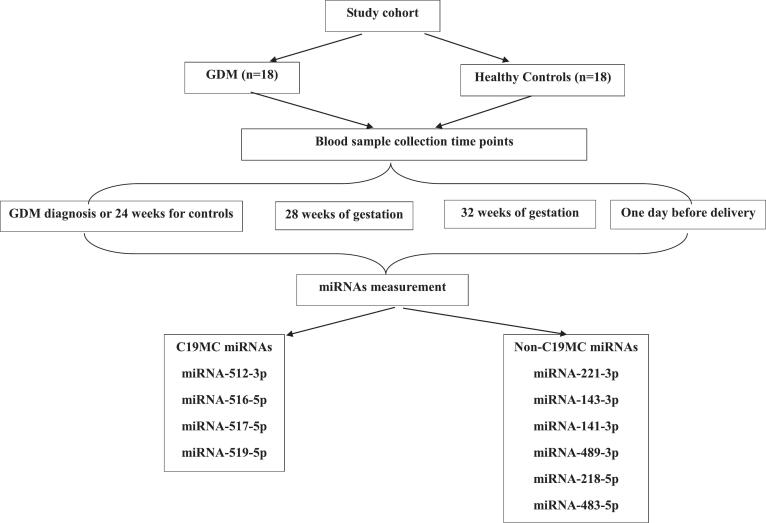
The flow chart of participants, sampling time points and miRNA measurements.

A commercial miRNA kit (cat#DP503, Tiangen Biotech, Beijing, China) was used for RNA extraction from serum and cDNA synthesis. Ten miRNAs were selected for measurement based on a recent study reporting a predictive effect on insulin sensitivity [14]. Significantly lower circulating levels of miRNA-221-3p and significantly increased circulating levels of the remaining nine miRNAs in the first trimester were reported to be associated with insulin sensitivity in the second trimester [14]. Of them, four miRNAs belong to C19MC miRNAs (miRNA-519d-5p, miRNA-512-3p, miRNA-516a-5p, and miRNA-517-5p), and six miRNAs belong to non-C19MC miRNAs (miRNA-141-3p, miRNA-143-3p, miRNA-218-5p, miRNA-221-3p, miRNA-483-5p, and miRNA-489-3p). The flow chart of participants, sampling time points and miRNA measurements is shown in Fig. 1.

The serum levels of these miRNAs at four time points were measured by quantitative real-time PCR using commercial kits (Cat#RR037A and RR420A, Takara, Japan). Primers of these nine C19MC miRNAs were purchased from Gene Adv Biology Company (Huzhou, China). Following the manufacturer's instructions, the RT-PCR conditions were 95 °C for 15 min, followed by 45 cycles of 94 °C for 20 s and 60 °C for 34 s. The results were normalised to the U6 level, which served as an internal control and was previously described [18], in each sample. All the measurements were conducted in duplicate.

### Statistical analysis

The serum levels of miRNAs were expressed as mean and standard deviation (SD) of 2^-ΔΔCT^. GraphPad Prism used the Mann-Whitney test to assess statistical differences between GDM and controls at each time point (10.1.2). *p* < 0.05 was considered significant.

## Results

The clinical details of women with GDM are summarized in Table 1. There was no statistical difference in maternal age, gravidity, parity, birthweight and delivery week between women with GDM and healthy controls. We first analysed the temporal dynamics of C19MC miRNAs across four gestational time points in women with GDM and healthy pregnancies. Previous studies have reported increased levels of these C19MC miRNAs in early gestation among women who later developed GDM [14]. As shown in Table 2, the relative expressions of miRNA-512-3p, miRNA-516a-5p, and miRNA-517-5p were significantly increased in GDM women at the time of diagnosis, at 28 weeks of gestation, and one day before delivery, compared to controls (*p* < 0.001). However, at 32 weeks of gestation, GDM women exhibited significantly lower expression levels of miRNA-512-3p and miRNA-517-5p than the control group. The increased expression levels of miRNA-516a-5p at 32 weeks of gestation were not different from controls (*p* = 0.2306). The relative expression of miRNA-519d-5p was significantly increased in GDM women at the time of diagnosis and at 28 weeks of gestation but significantly declined at 32 weeks and one day before delivery, compared to controls.

**Table 2:** The relative expressions of C19MC miRNAs in maternal circulation in GDM, across four time points.

miRNAs	GDM onset	28 weeks	32 weeks	Before delivery
miR-512-3p	1.582 ± 0.50 ^a^	6.320 ± 3.6 ^a^	0.831 ± 0.41 ^a^	1.837 ± 0.71 ^a^
miR-516a-5p	1.414 ± 0.59 ^a^	14.18 ± 9.8 ^a^	1.082 ± 0.59 ^b^	2.977 ± 1.58 ^a^
miR-517-5p	1.201 ± 0.32 ^a^	7.53 ± 4.4 ^a^	0.564 ± 0.18 ^a^	2.29 ± 0.99 ^a^
miR-519d-5p	2.106 ± 0.32 ^a^	1.111 ± 0.33 ^a^	0.787 ± 0.66 ^a^	0.571 ± 0.48 ^a^

Data were expressed as mean ± SD of 2^ ^(− ΔΔCT)^.

a: *p* < 0.01; b: *p* > 0.05 (GDM vs controls).

Next, we examined the temporal expression patterns of non-C19MC miRNAs in GDM and healthy pregnancies. miRNA-221-3p, previously reported to be downregulated in early gestation in women who later developed GDM [14]. As shown in Table 3, the relative expressions of miRNA-221-3p were significantly decreased at all four time points, GDM diagnosis, 28 and 32 weeks of gestation, and one day before delivery in GDM women, compared to controls.

**Table 3:** The relative expressions of non-C19MC miRNAs in maternal circulation in GDM, across four time points.

miRNAs	GDM onset	28 weeks	32 weeks	Before delivery
miR-221-3p	0.635 ± 1.067 ^a^	0.785 ± 1.51 ^a^	0.726 ± 1.07 ^a^	0.589 ± 1.21 ^a^
miR-143-3p	1.371 ± 2.06 ^a^	1.645 ± 3.11 ^a^	0.954 ± 1.34 ^b^	0.945 ± 1.19 ^b^
miR-141-3p	1.255 ± 1.56 ^b^	1.809 ± 1.51 ^b^	2.005 ± 3.13 ^b^	0.805 ± 0.91 ^a^
miR-489-3p	0.535 ± 0.307 ^a^	0.731 ± 0.36 ^a^	0.484 ± 0.26 ^a^	0.649 ± 0.49 ^a^
miR-218-5p	0.621 ± 0.381 ^a^	0.713 ± 0.57 ^a^	0.584 ± 0.48 ^a^	0.624 ± 0.35 ^a^
miR-483-5p	0.493 ± 0.484 ^a^	0.511 ± 0.60 ^a^	0.516 ± 0.537 ^a^	0.337 ± 0.35 ^a^

Data were expressed as mean ± SD of 2^ ^(− ΔΔCT)^.

a: p < 0.01; b: p > 0.05 (GDM vs controls).

The remaining five non-C19MC miRNAs, miRNA-143-3p, miRNA-141-3p, miRNA-489-3p, miRNA-218-5p, and miRNA-483-5p, have been reported to be upregulated in early gestation in women who later developed GDM [14]. As shown in Table 3, the relative expression of miRNA-143-3p was significantly increased at the time of diagnosis and at 28 weeks of gestation, but significantly declined at 32 weeks and 1 day before delivery. The relative expressions of miRNA-141-3p in GDM were not different from those in controls before delivery. In contrast, the relative expressions of miRNA-489-3p, miRNA-218-5p, and miRNA-483-5p were consistently decreased at all four time points in GDM women compared to controls.

## Discussion

**Fig. 2:**
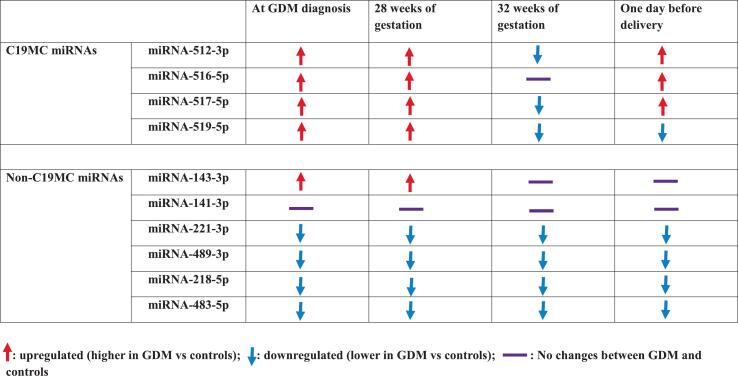
Summary of longitudinal changes in maternal circulating miRNAs in GDM across gestation, compared to controls.

In this longitudinal study aimed at validating previous findings, we found that three maternal circulating C19MC miRNAs (miRNA-512-3p, miRNA-516a-5p, and miRNA-517-5p) were consistently upregulated at the time of diagnosis, 28 weeks of gestation, and one day before delivery in GDM. C19MC miRNA-519d-5p was upregulated at the time of diagnosis and 28 weeks of gestation. Among non-C19MC miRNAs, miRNA-221-3p was consistently downregulated, while miRNA-143-3p was only upregulated at diagnosis and 28 weeks of gestation in GDM. miRNA-141-3p expression did not differ between GDM and controls before delivery. In contrast, miRNA-489-3p, miRNA-218-5p, and miRNA-483-5p were consistently downregulated in GDM. A summary of longitudinal miRNA changes is presented in Fig. 2.

Maternal circulating miRNAs have been proposed as potential early predictive biomarkers for GDM (reviewed in [3]). However, most existing studies on early prediction have assessed miRNA expression at a single time point, thereby limiting their understanding of temporal dynamics. Changes observed at one gestational stage in women who developed GDM later may reflect normal physiological adaptation rather than disease-specific alterations in pregnancy. Therefore, it potentially leads to false positives when applied to the general population. Placental development can significantly influence miRNA expression. In contrast, longitudinal studies enable the tracking of miRNA trajectories over time, providing a more accurate understanding of their role in disease progression and helping to distinguish between physiological and pathological changes.

In this longitudinal study, we selected four C19MC miRNAs and six non-C19MC miRNAs that have been previously reported to have early predictive value for GDM [14]. It is well known that placental dysfunction is associated with the development of GDM [19]. A large amount of placental material, such as extracellular vesicles, is released into the maternal circulation throughout gestation, contributing to maternal adaptation [20]. Measuring changes in C19MC miRNAs may therefore reflect both placental and metabolic changes. The four C19MC miRNAs (miRNA-512-3p, miRNA-516a-5p, miRNA-517-5p, and miRNA-519d-5p) we studied have been reported to be upregulated in early gestation in GDM [14]. Our findings confirmed the upregulated levels of these three miRNAs at diagnosis, 28 weeks, and before delivery, suggesting they are actively involved in the pathogenesis of GDM. Our findings reinforce their potential as early predictive biomarkers. These placenta-specific miRNAs contribute to maternal adaptation and immune modulation during pregnancy (reviewed in [9]). The observed decrease in these miRNAs at 32 weeks could be due to changes in placental EV release or maternal metabolic adaptation. However, our previous study suggested that the rate of EV production relative to placental weight remained unchanged [19], indicating that other regulatory mechanisms may be involved. With consistently elevated levels of C19MC miRNAs in GDM, our results support their utility for predicting ongoing placental dysfunction and disease progression, as reported in a previous study [14].

miRNA-221-3p has been shown to play a protective role in pancreatic β cells, specifically by regulating insulin secretion [21]. Downregulated levels of miRNA-221-3p were reported in GDM mouse models [21], and downregulated levels of miRNA-221-3p at early gestation were associated with insulin sensitivity between 24 and 29 weeks of gestation [14]. Our study found consistently downregulated levels of miRNA-221-3p across all time points in GDM, suggesting impaired insulin sensitivity and reinforcing its potential as a predictive biomarker for GDM.

Both miRNA-143-3p and miRNA-141-3p play important roles in insulin sensitivity. miRNA-143-3p specifically regulates genes associated with the insulin signalling pathway, including oxysterol-binding protein-related protein 8 (ORP8), which influences insulin sensitivity [22]. miRNA-143-3p may primarily regulate glucose uptake [23]. Our results showed that miRNA-143-3p was upregulated at diagnosis and at 28 weeks, but declined thereafter. Our results suggest that miRNA-143-3p may be involved in early metabolic dysfunction in GDM, but not necessarily in disease severity or progression. It may have a time-limited mechanistic role in GDM, although a previous study suggested it may have an early predictive effect [14]. Additionally, no change in circulating miRNA-141-3p levels between GDM and controls before one day of delivery, as observed in this study, may be due to sample variation.

In contrast to previous findings that reported upregulated levels of miRNA-489-3p, miRNA-218-5p, and miRNA-483-5p in early gestation in women who later developed GDM [14], our study showed consistently downregulated levels of miRNA-489-3p, miRNA-218-5p, and miRNA-483-5p across all time points in GDM. This discrepancy may reflect dynamic maternal and placental adaptations during the middle to late stages of gestation. This difference makes it difficult to determine whether the changes in these three non-C19MC miRNAs are causally related to GDM onset, reflect disease progression, or are simply a consequence of normal maternal or placental adaptation during pregnancy.

Several signalling pathways, including PI3K and mTOR pathways, play critical roles in the development of insulin resistance. Previous studies indicated that both C19MC miRNAs, such as miRNA-512-3p [24], and non-C19MC miRNAs, such as miRNA-141-3p and miRNBA-143-3p [25], [26], directly target components of these cascades. These findings suggest that miRNAs measured in this study may modulate insulin sensitivity by regulating PI3K/mTOR signalling.

We acknowledge several limitations. First, the absence of miRNA measurements during the first trimester limits our ability to assess early predictive value directly. However, selecting ten miRNAs that are known to be involved in insulin sensitivity and placental function is the first step in investigating temporal dynamics. This first step can help us identify potentially effective early predictive miRNAs. Second, the sample size was relatively small. Nonetheless, the strength of this study lies in its longitudinal design and biological depth. By analysing four key gestational time points per participant, we captured the temporal dynamics of these miRNAs across pregnancy.

In conclusion, our pilot longitudinal study supports the predictive potential of several miRNAs previously identified in early gestation, particularly the C19MC miRNAs (miRNA-512-3p, miRNA-516a-5p, and miRNA-517-5p) and the non-C19MC miRNA-221-3p. Other miRNAs require further validation. However, these findings warrant validation in the future with a large sample size and prospective studies to confirm their clinical utility as biomarkers for early prediction and monitoring of GDM. Our study suggests that longitudinal changes in these miRNAs contribute to the pathogenesis of GDM by regulating insulin sensitivity during pregnancy, and that measuring these miRNAs in early pregnancy may provide predictive value for GDM. Additionally, a better understanding of these miRNAs in regulating insulin sensitivity could lead to novel prevention or therapeutic strategies for GDM.

## CRediT authorship contribution statement

**Ying Gu:** Writing – original draft, Investigation, Conceptualization. **Yu Chen:** Investigation, Conceptualization. **Jingyang Li:** Data curation. **Lingli Hu:** Data curation. **Yajie Gao:** Resources. **Hua Gong:** Methodology. **Qi Chen:** Writing – review & editing, Writing – original draft, Investigation, Conceptualization.

## Consent for publication

N/A.

## Ethics statement

The Ethics Committee of Wuxi Maternity and Child Health Care Hospital, China approved this study (reference number 2023010628–16). Blood samples were collected from participants who provided signed consent.

## Funding

This study was funded by the Wuxi Healthy and Family Planning Commission, China (reference number: FYKY202101).

## Declaration of competing interest

The authors declare that they have no known competing financial interests or personal relationships that could have appeared to influence the work reported in this paper.
